# Ego Impairment Index (EII‐2) as a predictor of outcome in short‐ and long‐term psychotherapy during a 5‐year follow‐up

**DOI:** 10.1002/jclp.23332

**Published:** 2022-02-28

**Authors:** Jaakko Stenius, Erkki Heinonen, Olavi Lindfors, Juha Holma, Paul Knekt

**Affiliations:** ^1^ Department of Psychiatry Kuopio University Hospital Kuopio Finland; ^2^ Department of Forensic Psychiatry University of Eastern Finland, Niuvanniemi Hospital Kuopio Finland; ^3^ Department of Public Health and Welfare Finnish Institute for Health and Welfare Helsinki Finland; ^4^ Department of Psychology University of Oslo Oslo Norway; ^5^ Department of Psychology University of Jyväskylä Jyväskylä Finland

**Keywords:** ego functioning, Ego Impairment Index, psychotherapy, Rorschach, treatment planning

## Abstract

**Objective:**

This study examined the predictive ability of the Rorschach‐based Ego Impairment Index (EII‐2) on outcome of psychotherapy in different types and durations of therapy.

**Method:**

A total of 326 outpatients suffering from depressive or anxiety disorders were randomized into receiving solution‐focused (*n* = 97), short‐term psychodynamic (*n* = 101), or long‐term psychodynamic psychotherapy (*n* = 128). Psychotherapy outcome assessments during the 5‐year follow‐up period covered psychiatric symptoms, social functioning, and work ability.

**Results:**

Lower EII‐2 values, which indicate less problematic ego functioning, were found to predict faster improvement in both short‐term therapies as compared to long‐term psychotherapy.

**Conclusion:**

The results provide preliminary support for the utility of EII‐2 as a complementary measure to interview‐based methods for selecting between short‐ and long‐term therapies.

## 1 INTRODUCTION

Based on clinical experience, many psychotherapists have long considered that the choice of a psychotherapeutic approach should be adapted to the patient's individual characteristics to enhance treatment effectiveness. Accordingly, one of the most common reasons endorsed by psychologists for conducting psychological assessment is understanding the patient's unique psychological characteristics that may inform optimal selection and carrying out of therapeutic interventions (Wright et al., 2017). Indeed, recent psychotherapy research has yielded growing support for such views, showing that psychotherapy outcomes may be enhanced by considering various different patient characteristics beyond diagnoses (Norcross & Wampold, 2018). Certain patient qualities may also be relevant for optimal treatment length, that is, the amount of psychotherapy needed for a successful recovery (Alanne et al., 2021).

Often, suitability for a particular type or duration of psychotherapy has been thought to be best evaluated by an interviewer who is familiar with the model of treatment and its proposed indications and contraindications (Hoyt, 2011). However, little systematic comparative research exists on the patient characteristics that contribute to positive versus negative outcomes in different therapies and durations of treatment. Nevertheless, there is recent evidence that interview‐based assessments of intrapsychic and interpersonal qualities may be helpful in determining the duration and/or modality of psychotherapy to select for a given patient (Koelen et al., 2012). More specifically, qualities such as coherent self‐concept and better capacity for affect regulation have been shown to predict sufficiency of brief treatment (Laaksonen et al., 2013), whereas vulnerabilities in these (Laaksonen et al., 2013) and other personality structures (e.g., low‐level personality organization, lacking interpersonal capacities, and unstable identity) have been shown to predict inadequacy of short‐term psychotherapy and more likely need for long‐term psychotherapy (Knekt et al., 2017).

Many of the aforementioned qualities are captured by the concept of *ego functioning* (e.g., Auchincloss, 2015). Ego functioning comprises a variety of mature capacities, such as problem solving, interpersonal relatedness, affect regulation, and impulse control, that enable adaptive functioning when encountering the challenges inherent to everyday life; as such, ego functioning reflects accurate perception and integration of one's own mental processes and external events. Although it therefore appears both theoretically and clinically plausible, no prior research has directly investigated whether the level of ego functioning might be helpful in making an informed choice concerning optimal treatment length or type: such as, whether brief therapy is sufficient or long‐term therapy is indicated for recovery. There is also a lack of knowledge concerning how personality assessments other than those based on interviews predict therapy outcome. Although they are comprehensive, interviews can be subject to their own biases and limitations (Krishnamurthy et al., 2021). To ensure valid and reliable treatment decisions, it is therefore important to examine the predictive ability of other, complementary methods of assessing those psychological characteristics (e.g., personality structure, ego strengths and weaknesses) that may be relevant for treatment planning and selection.

The Rorschach Inkblot Method has been ranked as one of the most frequently used tools for psychological assessment in North America (Wright et al., 2017). As a performance‐based measure, it provides a different method from interview‐based approaches for evaluating dimensions of personality structure and dynamics (e.g., self‐perception, relatedness to other people, and affect regulation). Hence, this approach may have potential implications for treatment selection. The Rorschach provides a sample of behavior in an interpersonal and affectively tinged, yet standardized, context with no obvious social desirability or pathology cues. Therefore, it can provide insight into behavioral dispositions and related facets of ego functioning, such as reality testing and impulse control, which may not be as apparent in an interview‐based assessment. Supporting its potential usefulness, the most recent and comprehensive meta‐analyses of the Rorschach's validity in relation to both introspectively (e.g., self‐report) and externally (e.g., psychiatric diagnosis) assessed criteria comprised 25,795 participants in total (Mihura et al., 2013), and showed considerable validity for assessing perceptual and cognitive processes in particular. However, further research is still needed to establish the most useful Rorschach indicators and their clinical range: that is, for what kinds of treatment decisions and outcomes do they provide valuable, and potentially incremental, information on?

As one such promising indicator, the Rorschach‐based Ego Impairment Index (EII; Perry & Viglione, 1991; EII‐2; Viglione et al., 2003) rated from a person's responses to the Rorschach method, is a composite variable created specifically to target deficits in ego functions. In a meta‐analysis including 13 independent samples (total *N* = 1402), the EII demonstrated validity in assessing degree of personality disturbance, its overall weighted effect size with other measures of psychiatric severity being *r* = 0.29 and highly significant (*p* = 0.000002) (Diener et al., 2011). These measures of personality disturbance and psychiatric severity have, in turn, been shown to predict psychotherapy outcomes (Koelen et al., 2012), suggesting the potential of EII also for such prognostic uses. In addition, in their meta‐analysis, Diener et al. found that the EII demonstrated higher validity (*r* = 0.45) in capturing psychiatric severity rated by researchers (e.g., researcher ratings of ego impairment based on diagnoses or social competency) as compared to other sources of information, such as patient self‐reports (*r* = 0.10). The authors concluded that the extant evidence supports the potential utility of the EII as a performance‐based measure of global psychological impairment that can complement self‐report instruments measuring psychological impairment. Further, in a study designed to test the ability of the EII to predict treatment outcome, lower EII values (i.e., less problems in ego functioning) predicted a positive response to antidepressant treatment for patients with major depression (Perry & Viglione, 1991)—an expectable finding given that these patients evidenced less psychological impairment. Also, a study of children in intensive inpatient psychiatric treatment found higher EII‐2 values to predict worse long‐term treatment outcome (Stokes et al., 2003). In addition, a recent study (Stenius et al., 2018) found the EII‐2 to target different phenomena than an interview‐based assessment for adult patients, which in turn has been previously shown to predict the amount (i.e., short‐ vs. long‐term) of psychotherapy that patients needed to recover (Laaksonen et al., 2013). Taken together, these findings indicate the need for further research on the predictive ability of the EII‐2 in choosing between therapies of different lengths or type for adult patients, which is still lacking. In the present study, we therefore examined the predictive ability of EII‐2 in two short‐term (solution‐focused and psychodynamic) and one long‐term (psychodynamic) psychotherapy models in the treatment of depressive and anxiety disorders in adult outpatients. To evaluate the potential benefits of these treatment models in a sufficiently comprehensive manner—that is, to cover the central domains of mental health and functioning—we examined how EII‐2 scores predicted changes in psychiatric symptoms, work ability and social functioning during a 5‐year follow‐up period.

Based on previous empirical findings (Koelen et al., 2012; Laaksonen et al., 2013) that suggest better psychological resources to predict better treatment outcomes, we hypothesized better outcomes to be predicted by lower ego impairment within the different therapy types over the 5‐year follow‐up. Further, literature indicates that the better the psychological resources are for psychotherapy, the faster the gain is in short‐term therapies as compared to long‐term therapies (cf. Knekt et al., 2017; Laaksonen et al., 2013). Accordingly, we anticipated that when comparing different therapy groups, lower ego impairment would predict a faster symptom reduction in the short‐term therapy approaches relative to the long‐term therapy approach during the first year of the follow‐up period. Finally, we hypothesized that long‐term psychotherapy would benefit patients with greater ego impairment more than short‐term therapy in the long run.

## 2 SUBJECTS AND METHODS

The Helsinki Psychotherapy Study (HPS) (Knekt & Lindfors, 2004) is focused on studying the effectiveness and suitability of three forms of psychotherapy. More details on the design, patients and therapies can be found in Knekt and Lindfors (2004).

### 2.1 Patients

The patient sample included 326 outpatients from the Helsinki area (mean age = 32 years; range = 20–46) suffering from depressive or anxiety disorders. The patients were randomized into receiving solution‐focused (SFT) (*n* = 97), short‐term psychodynamic (SPP) (*n* = 101) or long‐term psychodynamic psychotherapy (LPP) (*n* = 128). The inclusion criteria for the present study further included that the duration of the disorder was at least 1 year and causing severe dysfunction in work ability. The subjects with psychotic, bipolar type I, severe personality, adjustment, substance abuse, or organic disorder, as well as intellectual disabilities, working within psychiatric health care, or having received psychotherapy within the previous 2 years were excluded from the study.

The study protocol was in accordance with the Declaration of Helsinki, and approval for the study was obtained from the ethics council of the Helsinki University Central Hospital. All of the patients provided written informed consent before entering the study.

### 2.2 Therapies

SFT is a short‐term resource‐oriented and goal‐focused therapeutic approach which helps clients change by constructing solutions and is based on an approach developed by de Shazer and colleagues (Hoyt, 2011). The frequency of sessions in SFT was flexible, usually once every 2–3 weeks, and the mean length of therapy was 7.5 months (SD = 3.0).

SPP is a focal, transference‐based therapeutic approach, which helps patients by exploring and working through specific intrapsychic and interpersonal conflicts. The orientation was based on approaches described by Malan and Sifneos (Hoyt, 2011). It was scheduled once a week and the mean duration of therapy was 5.7 months (SD = 1.3).

LPP is an intensive, transference‐based therapeutic approach which helps patients by exploring and working through a broad area of intrapsychic and interpersonal conflicts. The orientation followed the clinical principles of LPP (Gabbard, 2010). The frequency of sessions in LPP was 2–3 times a week and the mean duration of therapy was 31.3 months (SD = 11.9).

In this study, SFT was manualized and clinical adherence was monitored. Both psychodynamic therapies were conducted in accordance with clinical practice, with no monitoring.

### 2.3 Therapists

The therapies were provided by a total of 55 licensed psychotherapists. SFT was conducted by 6, SPP by 12, and LPP by 41 therapists (Knekt et al., 2017). The therapists delivering SPP and LPP had completed standard psychodynamic training lasting at least 3 years. Therapists practicing SPP had completed an additional short‐term training on psychodynamic therapy. The therapists providing SFT had been qualified in this practice by a local institute. All of the therapists had at least 2 years of postgraduate psychotherapy experience. The therapists had average psychotherapeutic work experience of 18 years (range = 6–30 years) in LPP, 16 years (range = 10–21) in SPP, and 9 years (range = 3–15) in SFT.

### 2.4 Assessments at baseline

The current study was conducted as a cohort study with repeated measurements: the patients were assessed at the baseline and after 3, 7, 12, 24, 36, 48, and 60 months (follow‐up period).

#### 2.4.1 Predictor variable

The Rorschach EII‐2, used as a predictor, was assessed at the baseline. The Rorschach was administered and coded according to procedures of the Rorschach Comprehensive System (CS) articulated by Exner (2003). The interrater agreement of coding has been published in detail previously (Valkonen et al., 2012).

The EII‐2 is a composite score of psychological impairment, as defined by deficits in ego functioning. The EII‐2 is derived from the following six CS variables: perceptual inaccuracy (*FQ‐*), disorganized language and thought (*WSum6*), the expression of primitive contents that are typically inhibited (*Critical Contents*), distortions in object representations (*M‐*), and *Good Human representation* (*GHR*) and *Poor Human representation* (*PHR*) variables reflecting adaptive versus problematic representations of people and interactions. As combined, *GHR* and *PHR* form a variable named *Human Representational Varible* (*HRV*) that summarizes data regarding object relations. *FQ‐* is coded from responses with poor match between the percept of the respondent and shape of a blot. *WSum6* is the weighted sum of six codes targeting various kinds of thought disturbance (e.g., strained reasoning and inappropriate integration of ideas). *Critical Contents* include *Anatomy*, *Blood*, *Fire*, *Explosion*, *Sex*, *X‐ray*, *Aggressive Movement*, and *Morbid* content responses. *M‐* is coded from human movement responses with poor form quality. *GHR* and *PHR* variables are based on an algorithm that combines data on the quality of responses with human content or interaction. The EII‐2 is calculated using particular weights for each of the six variables while controlling for *R*, the number of responses. The EII‐2 was divided by the median into two categories, “low” (indicating less ego impairment) and “high” (indicating greater ego impairment). The categorization was done to avoid the linearity assumption and two categories were used given the relatively small sample size that could result in reduced statistical power in the case of more categories. The median was used because of lack of a justified threshold value for EII‐2. CS summary scores from the protocols were calculated using the RIAP‐3 program. The EII‐2 score was derived from the summary scores using the Rorschach Research Utilities (RRU) program (Janson, 2008) and SPSS statistical software. The median and variance of the EII‐2 were −0.375 and 2.28, respectively. The interrater reliability of the EII‐2 was 0.85 demonstrating that coding of the EII‐2 was completed with good to excellent reliability. Previous studies on EII's reliability have demonstrated adequate test‐retest consistency both during a 9‐week follow up (Perry & Viglione, 1991) and a 5‐year follow‐up period (Perry et al., 1995).

#### 2.4.2 Other baseline measures

Psychiatric diagnoses were assessed according to the *DSM‐IV* criteria (American Psychiatric Association, 1994) based on a semistructured diagnostic interview (Knekt & Lindfors, 2004). Demographic background, psychiatric history and previous treatment were assessed via interviews and questionnaires. Psychiatric symptoms were assessed using the Beck Depression Inventory (BDI; Beck et al., 1961), while social functioning was assessed with the Life Situation Survey (LSS; Chubon, 1987). Personality functioning was evaluated with the AF score of the Structural Analysis of Social Behavior Introject Questionnaire (SASB, AF; Benjamin, 1996).

### 2.5 Psychotherapy outcome

Psychotherapy outcome was assessed via self‐reported psychiatric symptoms, work ability, and social functioning. General psychiatric symptoms and psychological distress were assessed using the Global Severity Index of the Symptom Checklist (SCL‐90‐GSI; Derogatis et al., 1973). The SCL‐90 was measured at baseline and seven times (3, 7, 12, 24, 36, 48, and 60 months) over a 5‐year follow‐up period. Work ability and functioning were evaluated using a modified Work Ability Index (WAI; Ilmarinen, 2019), which is a self‐reported measure that evaluates patients' capacities and resources at work. The WAI assessment was conducted at baseline and six time points (7, 12, 24, 36, 48, and 60 months) during the 5‐year follow‐up period. Finally, the global score of the self‐report inventory Social Adjustment Scale (SAS‐SR; Weissmann & Bothwell, 1976) was used to assess social functioning. The SAS‐SR was measured at baseline and seven times (3, 7, 12, 24, 36, 48, and 60 months) during the 5‐year follow‐up period. The reliability (Cronbach *α*) of each of the outcome measures, SCL‐90‐GSI, WAI, and SAS‐SR, was excellent or good, 0.96, 0.75, and 0.87, respectively.

### 2.6 Statistical methods

A cohort study design with repeated measurements was used. To analyze the prediction of the EII‐2 on psychotherapy outcome, the “intention‐to‐treat” (ITT) design was followed, in which all the patients who had been randomized were included. The analyses were based on the assumption of ignorable dropouts (Härkänen et al., 2005). The statistical analyses were performed using linear mixed models (Verbeke & Molenberghs, 1997). The dependent variables were the outcome measures (SCL‐90‐GSI, WAI, and SAS‐SR). The independent variables included the EII‐2 measured at baseline, therapy group, and the time of measurement during follow‐up, their first‐ and second‐order interactions, and a correction term (i.e., the difference between the theoretical and realized date of measurement due to some patients answering the questionnaires later, some earlier than others), along with seven potentially confounding factors (education, comorbidity of mood and anxiety disorder, separation experiences (i.e., significant early separations—or threat of separations—from caregivers), psychiatric symptoms [BDI], social functioning [LSS and SAS‐SR] and personality functioning [SASB, AF]) and the outcome measure at baseline. Model‐adjusted differences in outcomes between patients with “low” and “high” ego impairment (categories based on EII‐2 scores) at different measurement points were calculated (Lee, 1981) and the confidence intervals were computed using the delta method (Migon & Gamerman, 1999). The intraclass correlation coefficient (ICC) was calculated to evaluate interrater reliability between the two independent coders for the EII‐2 in 20 randomly selected protocols. The statistical analyses were performed with the SAS software, version 9.1 (SAS Institute Inc., 2007).

## 3 RESULTS

### 3.1 Baseline characteristics of the study population

The study group comprised 326 patients. The mean age of the patients was 32.3 years and 76.1% of them were women. Of the patients, 51.2% were single, with 25.8% having completed an academic education. A total of 84.6% of the patients were diagnosed with mood disorder, 43.6% had anxiety disorder, and 42.9% had comorbid psychiatric diagnoses. Regarding psychiatric treatment history, 19.3% of the patients had received psychotherapy previously, and 22.0% had used psychotropic medication. Apart from a lower prevalence of anxiety disorders in the long‐term psychotherapy group (36.7%) as compared to the short‐term psychodynamic (49.5%) and solution‐focused therapy (46.4%) groups, no statistically significant between‐group differences were noted in the baseline values of the predictor, outcome, or background variables of patients. For further patient details, see Alanne et al. (2021).

### 3.2 Comparison of outcome among EII‐2 groups within the therapy groups

Study of the changes during follow‐up in the outcome measures showed a significantly greater reduction for psychiatric symptoms (SCL‐90‐GSI) and improved social functioning (SAS‐SR) in the SFT group among individuals with higher EII‐2 reflecting greater ego impairment, but only at the 3‐month point, indicated by mean score differences of 0.21 (95% confidence interval [CI]) (0.03, 0.38) and 0.24 (0.11, 0.37) (Table 1), respectively. No significant differences between EII‐2 groups were detected within the SPP or LPP groups.

**Table 1:** Estimated mean values of the three outcome measures (SCL‐90‐GSI, WAI, and SAS‐SR) within treatment groups and differences in mean values (95% confidence intervals[CI]) during the 5‐year follow‐up according to the low (< median score) and high (> median score) values of ego impairment

		SFT			SPP			LPP		
		Mean^a^		Mean difference (95% CI)^b^	Mean^a^		Mean difference (95% CI) b	Mean^a^		Mean difference (95% CI) b
Outcome measure	Time (month)	Low	High		Low	High		Low	High	
SCL‐90‐GSI	0	1.22	1.37		1.16	1.36		1.28	1.29	
	3	1.10	0.95	**0.21 (0.03, 0.38)**	0.95	1.15	−0.12 (−0.29, 0.05)	1.14	1.09	0.05 (−0.11, 0.21)
	7	0.84	0.97	−0.09 (‐0.30, 0.13)	0.88	0.94	0.02 (−0.19, 0.23)	1.13	1.00	0.13 (−0.07, 0.33)
	12	0.80	0.96	–0.12 (−0.33, 0.09)	0.76	0.87	−0.03 (−0.23, 0.17)	1.06	0.90	0.15 (−0.03, 0.33)
	36	0.80	0.89	−0.03 (−0.29, 0.21)	0.83	0.87	0.08 (−0.16, 0.31)	0.76	0.69	0.06 (−0.15, 0.27)
	60	0.70	0.79	−0.04 (−0.27, 0.19)	0.67	0.73	0.04 (−0.19, 0.27)	0.67	0.70	−0.04 (−0.23, 0.16)
WAI	0	34.4	32.8		33.8	33.7		34.3	33.1	
	7	37.8	37.8	−0.36 (−2.97, 2.25)	38.1	37.9	−0.18 (−2.72, 2.37)	36.9	35.9	0.71 (−1.72, 3.13)
	12	38.6	36.9	1.35 (−1.46, 4.15)	38.4	36.9	1.10 (‐1.61, 3.80)	37.5	37.4	−0.26 (−2.84, 2.32)
	36	37.7	38.7	−1.58 (−4.81, 1.65)	37.5	37.4	−0.37 (−3.43, 2.70)	39.6	39.7	−0.51 (−3.40, 2.38)
	60	39.6	37.9	1.23 (−2.10, 4.55)	36.9	37.9	−1.58 (−4.87, 1.72)	40.7	39.7	0.68 (−2.19, 3.56)
SAS‐SR	0	2.23	2.20		2.16	2.15		2.20	2.17	
	3	2.24	1.95	**0.24 (0.11, 0.37)**	2.10	2.12	−0.04 (−0.16, 0.09)	2.17	2.14	−0.02 (−0.14, 0.11)
	7	2.04	1.96	0.02 (−0.14, 0.17)	1.99	1.98	−0.02 (−0.17, 0.13)	2.19	2.04	0.11 (−0.03, 0.25)
	12	2.00	1.97	−0.04 (−0.19, 0.11)	1.91	1.94	−0.05 (−0.20, 0.09)	2.11	1.96	0.11 (−0.03, 0.25)
	36	1.96	1.88	0.03 (−0.15, 0.20)	1.93	1.90	−0.004 (−0.16, 0.17)	1.89	1.79	0.07 (−0.08, 0.23)
	60	1.87	1.93	−0.12 (−0.29, 0.04)	1.86	1.81	0.03 (−0.13, 0.20)	1.83	1.80	−0.01 (−0.15, 0.14)

*Note*: Bold numbers indicate statistically significant differences between “low” and “high” categories of ego impairment. SFT, SPP and LPP respectively refer to solution‐focused therapy, short‐term psychodynamic psychotherapy and long‐term psychodynamic psychotherapy.

^a^ The model includes the confounding factors education, comorbidity of mood and anxiety disorder, separation experiences, BDI, LSS, SAS‐SR, and SASB, AF.

^b^ The model is further adjusted for baseline of respective outcome variable.

### 3.3 Comparison of outcomes between therapies within the EII‐2 groups

For the patients with lower EII‐2 values indicating lesser ego impairment, during the first year of the follow‐up period, SPP presented a more beneficial effect compared with LPP, showing more improved psychiatric symptoms (SCL‐90‐GSI) at the 3‐, 7‐, and 12‐month follow‐ups, the model‐adjusted mean differences being −0.17 (−0.34, −0.01), −0.23 (−0.43, −0.03), and −0.27 (−0.47, −0.08), respectively, and social functioning (SAS‐SR) at the 7‐ and 12 month follow‐ups, the mean differences being −0.21 (−0.35, −0.06) and −0.21 (−0.35, −0.07), respectively (Table 2). Similarly, over the course of the first year of the follow‐up period, LPP was outperformed by SFT at the 7‐ and 12‐month measurement points, indicated by more improved psychiatric symptoms in SFT, with mean differences of 0.27 (0.07, 0.48) and 0.24 (0.05, 0.44), and a more improved social functioning, with the mean differences of 0.19 (0.04, 0.34) and 0.16 (0.02, 0.30), respectively. Finally, the results regarding the work ability measure (WAI) contrasted what had been observed for the SCL‐90‐GSI and SAS‐SR results, as short‐term therapies (in comparison to LPP) did not lead to WAI improvements during the early stages of therapy. On the other hand, LPP yielded better WAI results than SPP over the 5‐year follow‐up point, the mean difference being −3.48 (−6.55, −0.41).

**Table 2:** Estimated mean value differences (95% confidence intervals [CI]) of the three outcome measures (SCL‐90‐GSI, WAI, and SAS) between therapy groups during the 5‐year follow‐up according to the low (< median score) and high (> median score) values of ego impairment

		EII‐2 low Mean difference (95% CI) a, b		EII‐2 high Mean difference (95% CI) a, b	
Outcome measure	Time (month)	SPP versus LPP	LPP versus SFT	SPP versus LPP	LPP versus SFT
SCL‐90‐GSI	0				
	3	**−0.17 (−0.34, −0.01)**	0.03 (−0.14, 0.19)	0.00 (−0.17, 0.17)	**0.18 (0.01, 0.35)**
	7	**−0.23 (−0.43, −0.03)**	**0.27 (0.07, 0.48)**	−0.12 (−0.33, 0.09)	0.05 (−0.15, 0.26)
	12	**−0.27 (−0.47, −0.08)**	**0.24 (0.05, 0.44)**	−0.09 (−0.28, 0.10)	−0.02 (−0.22, 0.17)
	36	0.12 (−0.11, 0.35)	−0.07 (−0.31, 0.16)	0.11 (−0.11, 0.33)	−0.17 (−0.40, 0.06)
	60	0.03 (−0.17, 0.25)	−0.06 (−0.28, 0.15)	−0.04 (−0.26, 0.17)	−0.07 (−0.27, 0.14)
WAI	0				
	7	1.77 (−0.68, 4.23)	−2.11 (−4.57, 0.35)	**2.66 (0.15, 5.17)**	−2.11 (−4.57, 0.35)
	12	1.48 (−1.12, 4.08)	0.34 (−2.34, 3.02)	0.12 (−2.55, 2.80)	0.34 (−2.34, 3.02)
	36	−1.63 (−4.61, 1.35)	0.90 (−2.13, 3.93)	−1.78 (−4.74, 1.20)	0.90 (−2.13, 3.93)
	60	**−3.48 (−6.55, −0.41)**	1.74 (−1.29, 4.77)	−1.22 (−4.33, 1.89)	1.74 (−1.29, 4.77)
SAS‐SR	0				
	3	−0.08 (−0.20, 0.04)	−0.03 (−0.15, 0.10)	−0.06 (−0.18, 0.07)	**0.23 (0.10, 0.35)**
	7	**−0.21 (−0.35, −0.06)**	**0.19 (0.04, 0.34)**	−0.08 (−0.23, 0.07)	0.09 (−0.05, 0.24)
	12	**−0.21 (−0.35, −0.07)**	**0.16 (0.02, 0.30)**	−0.04 (−0.19, 0.10)	0.01 (−0.13, 0.15)
	36	0.02 (−0.14, 0.18)	−0.03 (−0.19, 0.14)	0.09 (−0.07, 0.25)	−0.07 (−0.24, 0.09)
	60	0.03 (−0.12, 0.19)	0.01 (−0.15, 0.16)	−0.01 (−0.16, 0.15)	−0.11 (−.26, 0.04)

*Note*: Bold numbers indicate statistically significant differences between therapy groups. SFT, SPP, and LPP, respectively, refer to solution‐focused therapy, short‐term psychodynamic psychotherapy and long‐term psychodynamic psychotherapy.

^a^ The model includes the confounding factors education, comorbidity of mood and anxiety disorder, separation experiences at childhood, BDI, LSS, SAS‐SR, and SASB, AF.

^b^ The model is further adjusted for baseline of the respective outcome variable.

Somewhat smaller differences between therapy groups were observable in patients with higher EII‐2 values, exhibiting greater ego deficits. At the 3‐month follow‐up point, patients with more problematic ego functioning demonstrated more reduction in psychiatric symptoms (SCL‐90‐GSI) and more improved social functioning (SAS‐SR) in SFT than LPP, with the mean differences of 0.18 (0.01, 0.35) and 0.23 (0.10, 0.35), respectively. Patients with higher EII‐2 scores also experienced greater improvements in work ability from SPP than LPP at the 7‐month follow‐up time point, with the mean score difference being 2.66 (0.15, 5.17); furthermore, patients with greater ego deficits (i.e., higher EII‐2 scores) did not benefit significantly more from LPP than SFT or SPP.

## 4 DISCUSSION

To clarify the predictive ability of the Rorschach‐based assessment of ego impairment on treatment process and effectiveness, we examined for the first time the prediction of the EII‐2 on psychotherapy outcome in two short‐term (psychodynamic and solution‐focused) versus one long‐term (psychodynamic) psychotherapy. In contrast to our hypotheses, the present study did not provide evidence that lower ego impairment, in comparison to greater ego impairment, predicts better outcome across the different therapy types, nor that long‐term treatment will yield better long‐term outcomes among patients with high ego impairment than short‐term therapy. However, lesser impairment was found to predict faster response in short‐term than long‐term psychotherapy during the first follow‐up year, as hypothesized and in line with previous empirical studies (e.g., Laaksonen et al., 2013), which have demonstrated better psychological resources and capacities to predict faster gain in short‐term therapy. Hence, these findings demonstrate the potential of the EII‐2 in identifying psychological characteristics that indicate capacity to benefit from short‐term treatment.

### 4.1 Patients with lower EII‐2 values

As hypothesized, lower ego impairment predicted faster improvement in SPP than LPP in terms of psychiatric symptoms (at the 3‐, 7‐ and 12‐month measurement points) and social functioning (at the 7‐ and 12‐month measurement points), as well as in SFT than LPP at the 7‐ and 12‐month measurement points. These findings are in line with previous studies of the same population that have reported faster reduction of psychiatric symptoms in short‐ than long‐term psychotherapy for patients with better interview‐rated psychological suitability (e.g., capacities for self‐reflection, affect tolerance, and flexible interaction) (Laaksonen et al., 2013) and personality functioning (Knekt et al., 2017). Likewise, the results of the present study fit with findings that lower EII‐2 values (i.e., lower ego impairment) predict greater symptomatic improvement in antidepressant treatment (Perry & Viglione, 1991).

Our findings suggest that characteristics of lower ego impairment, for example, capacity to establish mutually supportive relationships, regulate affect states, control impulses, and cope with frustration and anxiety, are particularly beneficial for gaining benefits from short‐term therapies. In SFT, patients with lower ego impairment scores may be able to readily construct alternative solutions to their problems, which is in line with the therapeutic approach of SFT (Hoyt, 2011). Similarly, patients who benefit from SPP may require adequate tolerance to anxiety and intense affects due to the deliberate focus on patients' core conflicts (Hoyt, 2011). Further, as SPP and SFT were both designed to achieve therapeutic changes within a limited time frame, ego functions—such as capacity to adaptively regulate behavior—may be critical to therapeutic outcome. Previous research has found EII‐2 to be relatively weakly associated with both self‐report and interview‐based methods of pretreatment assessment (e.g., Stenius et al., 2018), which is in line with the meager associations between Rorschach and introspection‐based measures, more generally (e.g., Mihura et al., 2013). Given this, the present findings support the incremental utility of the EII‐2 in selecting between short‐ and long‐term psychotherapy. One could surmise that due to the nature of Rorschach as a performance‐based problem‐solving situation that engages cognitive processes (e.g., perception, decision making, and logical reasoning), it may be potentially useful in assessing ego functions difficult to report verbally (e.g., reality testing and stress tolerance). Rorschach may therefore have utility alongside interviews in determining an optimal length of psychotherapy needed for recovery: as, for instance, in the case of a patient who demonstrates adequate adjustment on the surface, yet the interviewer suspects some underlying psychological weakness or disruption.

In contrast to the outcomes measured in terms of psychiatric symptoms and social functioning, virtually no significant differences in work ability, either within or between therapies, were observed among patients differing in ego functioning. One possible explanation for this finding is that work ability may be largely determined by factors, which cannot be directly targeted by psychotherapy—such as the patient's physical health and work‐place characteristics—at least for those patients who are psychologically relatively well‐functioning. This finding and interpretation is also in line with previous studies indicating smaller and slower improvements in work ability than psychiatric symptoms after various short‐ and long‐term treatments (Mintz et al., 1992).

### 4.2 Patients with higher EII‐2 values

In contrast to our hypothesis, greater ego impairment predicted faster improvement in psychiatric symptoms and social functioning in the SFT treatment group at the first (3‐month) follow‐up point, whereas no differences between patients with lower and greater ego impairment were found in the SFT group for subsequent follow‐up time points. It should also be noted that greater ego impairment predicted faster improvement in terms of psychiatric symptoms and social functioning in SFT than LPP at the 3‐month follow‐up point. However, this difference between SFT and LPP for patients with high EII‐2 scores disappeared during later time points.

SFT is a strength‐based, solution‐building approach, which concentrates on human resources and capacities (Hoyt, 2011). For instance, patients are encouraged to identify problem‐solving strategies that were successful in the past and present, as well as find alternatives to current patterns of behavior and interaction. Our findings may therefore reflect that patients who suffered from more problematic ego functioning—associated possibly with poor sense of one's agency (Eagle, 2020) and greater need for support and guidance from the therapist—may have experienced the encouragement and emphasis on personal strengths provided during SFT as highly beneficial at the start of therapy. In other words, the therapy was immediately useful for the client, unlike the more traditional discourse provided by the psychodynamic approach. Hence, the early benefits of SFT may have reflected the mobilization of the patient's positive expectations.

With respect to changes in patients' work abilities, LPP showed somewhat slower benefits than the two short‐term therapies for patients with greater ego impairment. These results suggest that the active, focused, and structured therapeutic approach utilized in short‐term treatments was particularly beneficial for patients with vulnerabilities in their ego functions. It is possible that this approach immediately enhanced their agency and self‐evaluated work ability.

Taken together, it seems a noteworthy and novel finding that patients with more problematic ego functioning may gain faster early benefits from short‐term as opposed to long‐term psychotherapy in some areas of functioning. One explanation for this finding is that patients' ego weaknesses may be associated with a need for the therapists to provide a function that patients lack (e.g., soothing, advice, encouraging, and self‐understanding). It could be postulated that these elements are not as actively provided by therapists at the initiation of long‐term psychodynamic psychotherapy. Hence, the structured, active, and clearly focused short‐term therapies seem to have been experienced as more helpful by patients, with support from the therapist perceived as more available through intense interaction and collaboration with the therapist. However, this effect was found to disappear by the subsequent follow‐up points, possibly reflecting a decreased need for therapist‐provided external structure as the patient's internal capacities develop during long‐term therapy.

Indeed, no notable between‐therapy differences in psychiatric symptoms and social functioning were observed after both short‐ and long‐term therapies had ended (i.e., during the 3–5‐year follow‐up period). Nevertheless, a statistically significant improvement in psychiatric symptoms and social functioning was detected in both “low” and “high” EII‐2 groups for LPP, but not in the short‐term therapies, during the 12–36‐month follow‐up period. This provides some support for the hypothesis that patients with more problematic ego functioning would benefit more from long‐term psychotherapy, as it might provide the appropriate conditions for strengthening and repairing ego weaknesses and vulnerabilities along with psychological capacities and skills. The minor outcome differences between the short‐term therapies and LPP may nevertheless be related to the decision to exclude patients with severe psychiatric disorders (e.g., psychoses and severe personality disorders) from the study. Thus, the presented results should not be generalized to indicate the prognostic validity of EII in such populations of patients.

### 4.3 Limitations

While the present study possessed a number of strengths—namely, a relatively large sample, long‐follow‐up period, and outcome assessments across several central domains of functioning—certain limitations should also be noted. First, the psychodynamic therapies were neither manualized nor monitored for adherence. However, it is important to note that this procedure is in line with normal clinical practice, which was the focus of our investigation. Second, all the outcome measures were based on patients' self‐reports; thus, the results convey only one, albeit important, perspective of the investigated subject. Third, since patients with psychotic or bipolar type I disorder, severe personality pathology, substance abuse, and cognitive impairment were excluded, the results might not be generalizable to depressed or anxious patients with these comorbidities. Although the EII is often used for evaluating patient populations with severe psychiatric disorders, it has also been posited to be sensitive to impairments in relatively well‐functioning individuals (Viglione et al., 2003), which was a reason for investigating its predictive value in the present study. However, if patients with severe personality disorder (low functioning patients) had been included in the study, presumably the contrast between low versus high functioning patients and, correspondingly, the predictive ability of the EII‐2 would have been even greater. Nevertheless, as the present investigation was designed to examine whether the EII‐2 is associated with the outcome of psychotherapies of different lengths and types, further research in appropriate designs is needed to determine the most useful clinical cutoff scores helpful for treatment choice for particular contexts and populations. Relatedly, it needs to be noted that in addition to psychotherapy approaches investigated in the present study, other well‐documented evidence‐based approaches for depression and anxiety, most notably cognitive‐behavioral psychotherapy, exist; hence, additional research might be considered also for clarifying the EII's potential predictive utility in these approaches. In addition, future research should investigate the potential incremental utility of the EII‐2 in conjunction with other measures, to establish their optimal conjoint predictive ability for optimal treatment selection. Finally, it should be noted that even though the findings were controlled for several known confounders, the possibility of residual confounding cannot fully be excluded.

## 5 CONCLUSIONS

According to the results of the present study, patients with lower EII‐2 values, which are indicative of lesser ego impairment, may be successfully treated with short‐term therapies. The results thus provide empirical support for the theoretical and clinical notions that ego functioning is relevant when considering which duration of therapy is appropriate for a patient. Similarly, the findings support the potential utility of the EII‐2, alongside interview‐based evaluations (e.g., Suitability for Psychotherapy Scale), in making such determinations.

However, as this was the first study to directly evaluate the predictive validity of EII‐2 on psychotherapy outcome in an adult population, further research should confirm and extend the presented findings.

### PEER REVIEW

The peer review history for this article is available at https://publons.com/publon/10.1002/jclp.23332.

## Data Availability

The data of the study are owned by the Finnish Institute for Health and Welfare. This is made available for researchers by request and with consent of the Helsinki Psychotherapy Study management board at the Finnish Institute for Health and Welfare regarding specific research questions. The data set cannot be publicly available due to privacy or ethical restrictions.
